# Comprehensive pharmacovigilance of phytoalkaloid chemotherapeutics: Signal detection and time-to-onset analysis based on FAERS

**DOI:** 10.1097/MD.0000000000046044

**Published:** 2025-11-21

**Authors:** Bangguo Song, Yang Zhang, Li Wang, Jihong Hu

**Affiliations:** a Clinical College of Chinese Medicine, Gansu University of Chinese Medicine, Lanzhou, China; b Key Laboratory of Dunhuang Medicine, Ministry of Education, Gansu University of Chinese Medicine, Lanzhou, China; c Research Center of Traditional Chinese Medicine, Gansu Province, Gansu University of Chinese Medicine, Lanzhou, China; d Scientific Research and Experimental Center, Gansu University of Chinese Medicine, Lanzhou, China; e College of Public Health, Gansu University of Chinese Medicine, Lanzhou, China; f Laboratory and Simulation Training Center, Gansu University of Chinese Medicine, Lanzhou, China.

**Keywords:** adverse drug events, disproportionality analysis, FAERS, pharmacovigilance, plant alkaloid chemotherapy, Weibull survival analysis

## Abstract

Plant alkaloid-based chemotherapeutic agents, including paclitaxel, vincristine, and irinotecan, play a crucial role in cancer treatment. However, their use is frequently associated with adverse drug events (ADEs), which can impact patient safety and treatment outcomes. Despite prior research, there is still a lack of comprehensive real-world data analysis to systematically investigate ADEs associated with these drugs. This study extracted ADE reports related to paclitaxel, vincristine, and irinotecan from the FDA Adverse Event Reporting System (FAERS) database up to Q4 2024. Data processing was conducted using MySQL and Statistical Analysis System software, and adverse events (AEs) were categorized based on the Medical Dictionary for Regulatory Activities (MedDRA) classification system. Disproportionality analysis was employed using 4 methodologies – reporting odds ratio (ROR), proportional reporting ratio, Bayesian confidence propagation neural network, and Multi-Item Gamma Poisson Shrinker (MGPS) – to detect statistically significant safety signals. Additionally, Weibull survival analysis was conducted to evaluate the time-to-onset distribution of ADEs, allowing for a deeper understanding of the temporal patterns of adverse reactions. A total of 31,007, 7389, and 12,049 ADE reports were retrieved for paclitaxel, vincristine, and irinotecan, respectively. Disproportionality analysis identified significant ADE signals across 27 system organ classes, with the most frequently reported AEs involving hematologic, gastrointestinal, neurological, and hepatic disorders. Novel ADE signals were detected for each drug: paclitaxel (10 new signals), vincristine (10 new signals), and irinotecan (8 new signals). Representative newly identified ADEs include dyspnea, flushing, and back pain for paclitaxel, febrile neutropenia, pancytopenia, and sepsis for vincristine, and neuropathy peripheral, malignant neoplasm progression, and pulmonary embolism for irinotecan. Time-to-onset analysis indicated that ADE occurrences predominantly peaked within the first 30 days of treatment, following an early failure pattern, suggesting that intensive monitoring during this period may be necessary. This study provides a comprehensive real-world safety evaluation of plant alkaloid-based chemotherapeutic agents, identifying both known and previously unreported ADEs. By leveraging large-scale FAERS data, multiple signal detection methodologies, and Weibull survival analysis, this research enhances the pharmacovigilance landscape, offering crucial insights for clinicians and regulatory authorities.

## 1. Introduction

Cancer remains one of the leading public health concerns worldwide, with chemotherapy serving as a crucial therapeutic strategy. Plant alkaloid-based chemotherapeutic agents, such as paclitaxel, vincristine, and irinotecan, have been widely utilized in the treatment of various malignancies, including breast cancer, lung cancer, colorectal cancer, and leukemia, owing to their unique antitumor mechanisms.^[1–3]^ These drugs function primarily by disrupting microtubule dynamics, thereby inhibiting cancer cell division and proliferation, effectively delaying tumor progression and improving patient survival outcomes.^[4]^ However, while these agents exert potent anticancer effects, they are also associated with a range of adverse drug events (ADEs), which may compromise patient tolerance and therapeutic efficacy.

According to the latest global cancer statistics, an estimated 20.9 million new cancer cases and 9.7 million cancer-related deaths occurred worldwide in 2024, underscoring the urgent need for effective chemotherapeutic strategies.^[5]^ Among the various classes of anticancer drugs, plant alkaloid–derived agents such as paclitaxel, vincristine, and irinotecan have become cornerstones of modern chemotherapy, being widely recommended in international guidelines as first-line or adjuvant regimens for breast, ovarian, hematologic, and colorectal malignancies. These agents exert antitumor activity mainly through the disruption of microtubule dynamics or inhibition of topoisomerase I, thereby blocking cancer cell proliferation. However, despite their proven efficacy, they are frequently associated with clinically significant ADEs. For instance, paclitaxel has been linked to severe neurotoxicity, hypersensitivity reactions, and bone marrow suppression, which restrict its long-term application.^[6]^ Vincristine, primarily used in acute lymphoblastic leukemia and lymphoma, often causes peripheral neuropathy and hematologic toxicity, compromising patients’ quality of life.^[7]^ Irinotecan, a standard therapy for colorectal cancer, is limited by gastrointestinal toxicity (notably diarrhea), bone marrow suppression, and hepatotoxicity.^[8]^ Therefore, a comprehensive analysis of ADEs associated with these representative plant alkaloid-based chemotherapeutic agents is essential for optimizing treatment regimens and ensuring patient safety.

The U.S. Food and Drug Administration (FDA) Adverse Event Reporting System (FAERS) is one of the largest global pharmacovigilance databases, compiling adverse event (AE) reports from healthcare institutions, pharmaceutical companies, and patients, making it a critical tool for post-marketing drug safety evaluation. However, the FAERS database presents inherent limitations, including variable data quality, reporting bias, and unclear causal relationships. Thus, advanced data mining techniques are required to enhance the reliability of ADE signal detection.^[9]^

In this study, we utilized the FAERS database to investigate ADEs associated with 3 widely used plant alkaloid-based chemotherapeutic agents: paclitaxel, vincristine, and irinotecan. Multiple analytical approaches were employed for ADE signal detection, including disproportionality analysis methods such as the reporting odds ratio (ROR) and proportional reporting ratio (PRR), as well as Bayesian confidence propagation neural networks (BCPNN) and the multi-item gamma Poisson shrinker (MGPS). Additionally, Weibull survival analysis was utilized to assess the time-to-onset distribution of ADEs, providing a deeper understanding of their occurrence patterns. The primary objectives of this study were as follows: To identify common ADEs associated with paclitaxel, vincristine, and irinotecan. To detect potential novel ADE signals and provide safety alerts for clinical practice. To analyze the time-dependent occurrence patterns of ADEs and offer precise risk management recommendations for clinical medication use.

## 2. Materials and methods

### 2.1. Data source

The ADE data used in this study were obtained from the FAERS database, which has been publicly accessible since 2004 for collecting AE reports from various sources, including healthcare professionals, pharmaceutical manufacturers, and patients. To investigate ADEs associated with the plant alkaloid-based chemotherapeutic agents paclitaxel, vincristine, and irinotecan, data were extracted from the FAERS database up to the fourth quarter (Q4) of 2024. The extracted data were subsequently imported into MySQL 15.0 and processed using Navicat Premium 15 software to facilitate comprehensive analysis. This study was conducted using data from the publicly available FAERS. All data are de-identified, and no direct patient contact was involved. Therefore, ethical approval and informed consent were not required in accordance with institutional policies and international guidelines.

### 2.2. Data extraction and analysis

**Figure 1.**
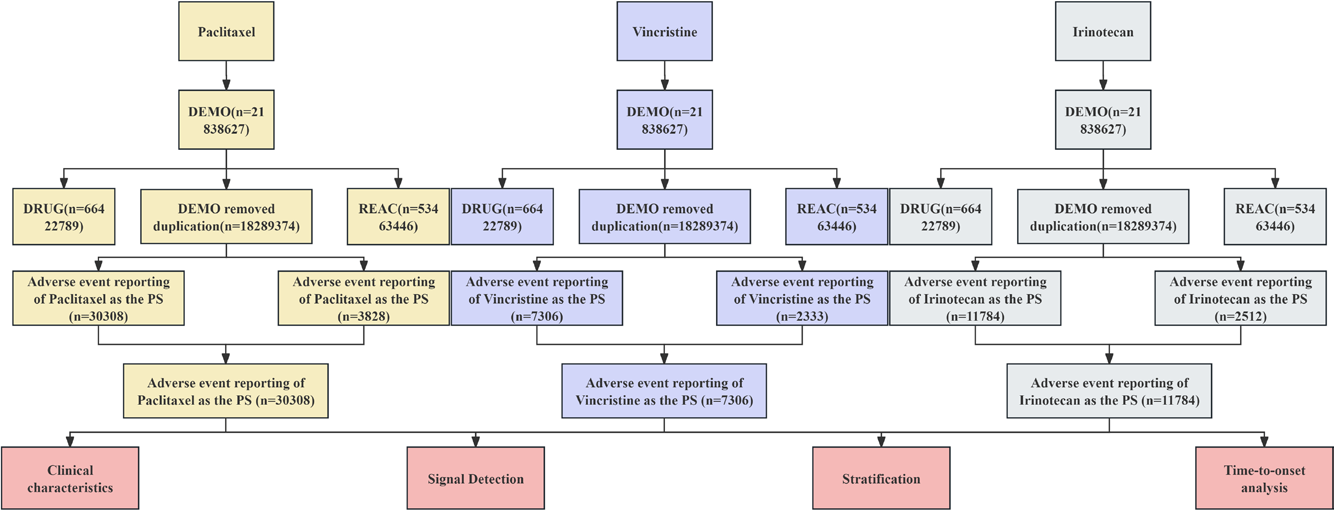
Workflow of the analysis of adverse drug events (ADEs) associated with paclitaxel, vincristine, and irinotecan. The diagram illustrates the overall process of data extraction, cleaning, MedDRA coding, signal detection, and time-to-onset analysis.

In this study, paclitaxel, vincristine, and irinotecan were designated as suspected drugs, with their names encoded using Medex_UIMA_1.8.3. The data obtained from the FAERS database were preprocessed using the Statistical Analysis System (SAS) and MySQL to ensure data integrity. Duplicate reports with the same case ID were removed from the DEMO table. Additionally, the latest version of the Medical Dictionary for Regulatory Activities (MedDRA 27.0) was utilized to match the preferred terms (PT) and corresponding SOC related to ADEs associated with paclitaxel, vincristine, and irinotecan. Clinical characteristics of patients who experienced ADEs related to these drugs were collected, including age, gender, body weight, reporting region, indications, and reporting time. The entire workflow for data extraction, processing, and analysis is illustrated in Figure 1.

### 2.3. Data mining algorithms

To identify potential associations between paclitaxel, vincristine, irinotecan, and ADEs, a disproportionality analysis was conducted. This analysis is considered a key pharmacovigilance tool and aims to evaluate the relationship between drugs and ADEs by comparing the observed frequency ratios in exposed and nonexposed populations using 2 × 2 contingency tables. In this study, 4 disproportionality methods were employed to detect ADE signals: ROR,^[10]^ PRR,^[11]^ BCPNN,^[12]^ and multi-item gamma Poisson shrinker (MGPS).^[13]^ Each of these methods has distinct advantages. ROR is beneficial in correcting biases caused by a small number of reported events. PRR offers higher specificity compared to ROR. BCPNN excels in integrating multi-source data and performing cross-validation. MGPS is particularly effective in detecting signals arising from rare events. The formulas and threshold values for these 4 algorithms are detailed in Table S1 (Supplemental Digital Content, https://links.lww.com/MD/Q713). Statistical analysis was performed using R software, where higher values indicate stronger signal intensity, suggesting a stronger association between the target drug and the ADE.

## 3. Results

### 3.1. Demographic characteristics of patients with AEs related to plant alkaloid chemotherapeutic agents

From the inception of the FAERS database until the fourth quarter of 2024, a total of 31,007 AE reports were recorded for paclitaxel, 7389 for vincristine, and 12,049 for irinotecan. Overall, demographic characteristics showed notable differences across the 3 agents.

Gender distribution revealed that paclitaxel was more frequently reported in female patients (63.5%), whereas vincristine (45.9%) and irinotecan (45.9%) were predominantly reported in male patients. In terms of age, the 18 to 65 year group constituted the largest proportion of cases for all 3 drugs, though the percentage was highest for paclitaxel (45.7%), moderate for irinotecan (36.7%), and lowest for vincristine (26.7%). Weight information was frequently missing (48.5%–72.3% across drugs); among available data, paclitaxel cases clustered in the 70 to 89 kg range, vincristine in < 50 kg, and irinotecan in 50 to 69 kg.

The primary treatment indications also reflected the distinct clinical use of these drugs: paclitaxel was often associated with breast and ovarian cancers but had a high proportion of “unknown indications” (23.9%); vincristine was most commonly linked to acute lymphocytic leukemia (18.1%); and irinotecan was mainly prescribed for metastatic colorectal cancer (15.1%).

Geographic distribution of reports further emphasized drug-specific trends. Paclitaxel reports were concentrated in the United States (23.9%), with additional contributions from France (8.2%) and Italy (3.4%). Vincristine reports came primarily from the United States (23.6%), France (17.7%), and Canada (15.4%). For irinotecan, the United States (22.0%), Japan (12.4%), and France (10.6%) were the leading reporting countries. Detailed demographic and clinical characteristics are summarized in Table 1.

**Table 1:** Clinical characteristics of paclitaxel, vincristine, and irinotecan adverse event reports from the FAERS database (storage time – Q4 2024).

Characteristics	Paclitaxel		Vincristine		Irinotecan	
Number of events	31,007		7389		12,049	
Gender, number (%)						
Female	19,699 (63.5%)		2745 (37.1%)		3984 (33.1%)	
Male	8304 (26.8%)		3391 (45.9%)		5466 (45.4%)	
Miss	3004 (9.7%)		1253 (17.0%)		2599 (21.6%)	
Age, number (%)						
Median (IQR)	62		21		63	
<18	115 (0.4%)		2761 (37.4%)		445 (3.7%)	
18–65	14,185 (45.7%)		1973 (26.7%)		4417 (36.7%)	
65–85	10,211 (32.9%)		1106 (15.0%)		3705 (30.7%)	
>85	126 (0.4%)		31 (0.4%)		31 (0.3%)	
Miss	6370 (20.5%)		1518 (20.5%)		3451 (28.6%)	
Weight (kg), number (%)						
<50	1225 (3.95%)		1042 (14.10%)		496 (4.12%)	
50–69	5737 (18.50%)		314 (4.25%)		1665 (13.82%)	
70–89	7284 (23.49%)		466 (6.31%)		1436 (11.92%)	
≥90	1718 (5.54%)		228 (3.09%)		563 (4.67%)	
Miss	15,043 (48.51%)		5339 (72.26%)		7889 (65.47%)	
Top 5 indication, number (%)	Product used for unknown indication	28,657 (23.9%)	Acute lymphocytic leukaemia	7154 (18.1%)	Colorectal cancer metastatic	7597 (15.1%)
	Breast cancer	9815 (8.2%)	Diffuse large B-cell lymphoma	3632 (9.2%)	Product used for unknown indication	6664 (13.3%)
	Pancreatic carcinoma	4108 (3.4%)	Product used for unknown indication	2797 (7.1%)	Pancreatic carcinoma	3037 (6.1%)
	Ovarian cancer	3869 (3.2%)	Non-Hodgkin Lymphoma	2025 (5.1%)	Colorectal cancer	2396 (4.8%)
	Pancreatic carcinoma metastatic	3613 (3.0%)	B-cell lymphoma	1929 (4.9%)	Colon cancer	2132 (4.2%)
Top 5 reported countries, number (%)	United States	28,657 (23.9%)	United States	1747 (23.6%)	United States	2648 (22.0%)
	France	9815 (8.2%)	France	1307 (17.7%)	Japan	1490 (12.4%)
	Italy	4108 (3.4%)	Canada	1137 (15.4%)	France	1273 (10.6%)
	Japan	3869 (3.2%)	Italy	765 (10.4%)	Italy	1127 (9.4%)
	Germany	3613 (3.0%)	United Kingdom	452 (6.1%)	United Kingdom	741 (6.1%)

IQR = interquartile range.

**Figure 2.**
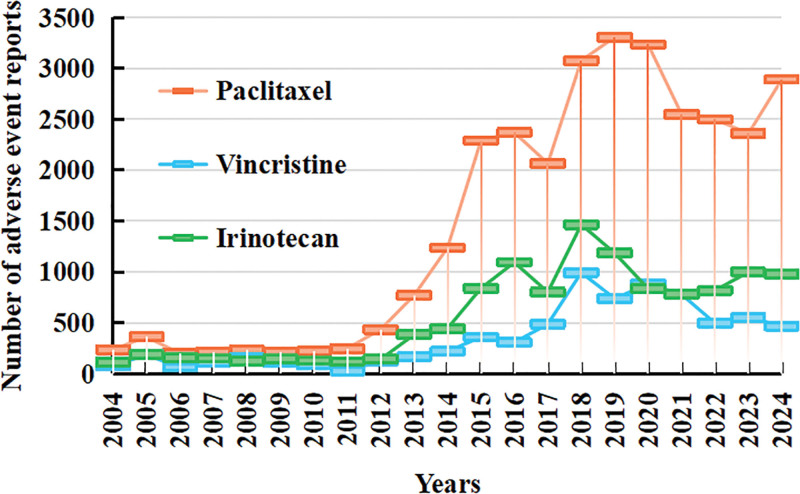
Temporal trends in adverse event (AE) reporting for paclitaxel, vincristine, and irinotecan from the FAERS database (2004–2024). The figure highlights the rising trend of ADE reports in recent years, with drug-specific differences in reporting frequency. ADE = adverse drug events.

Figure 2 illustrates the number of AE reports associated with paclitaxel, vincristine, and irinotecan over time. Between 2004 and 2016, the number of reported ADEs for all 3 drugs showed a steady increase. From 2017 to 2019, the growth rate accelerated, followed by a decline between 2019 and 2022. In 2023, the number of reports began to rise again. Over the past 5 years, the average annual number of reports exceeded 2700 for paclitaxel, 600 for vincristine, and 800 for irinotecan. Figure 2 provides a detailed visualization of these trends.

### 3.2. Signal detection at the system organ class (SOC) level

Statistical analysis identified 27 system organ classes (SOCs) associated with AEs linked to paclitaxel, vincristine, and irinotecan (Table 2). Among them, paclitaxel showed positive safety signals in 11 SOCs, including gastrointestinal, skin and subcutaneous tissue, immune system, blood and lymphatic, respiratory, vascular, cardiac, metabolic and nutritional, neoplasms (benign, malignant, and unspecified), hepatobiliary, and endocrine disorders. Vincristine demonstrated signals in 9 SOCs, most prominently congenital, familial and genetic disorders; blood and lymphatic disorders; nervous system disorders; infections and infestations; hepatobiliary and endocrine disorders; as well as neoplasms and ear/labyrinth disorders. Irinotecan was associated with 8 SOCs, notably gastrointestinal, infections, respiratory, blood and lymphatic, metabolic and nutritional, hepatobiliary, vascular, and neoplasms (benign, malignant, and unspecified).

**Table 2:** Signal strength of paclitaxel, vincristine, and irinotecan AEs across system organ classes (SOC) in the FAERS database.

SOC	Paclitaxel		Vincristine		Irinotecan	
	N	ROR (95% Cl)	N	ROR (95% Cl)	N	ROR (95% Cl)
Blood and lymphatic system disorders	6388	**4.16 (4.05–4.26**)	3105	**10.64 (10.24–11.05**)	3049	**5.03 (4.85–5.22**)
Cardiac disorders	3173	**1.27 (1.23–1.32**)	490	0.93 (0.85–1.01)	777	0.77 (0.72–0.82)
Congenital, familial and genetic disorders	132	0.45 (0.38–0.54)	106	**1.75 (1.44–2.11**)	62	0.53 (0.42–0.68)
Ear and labyrinth disorders	246	0.60 (0.53–0.68)	123	**1.43 (1.20–1.71**)	35	0.21 (0.15–0.30)
Endocrine disorders	340	**1.40 (1.26–1.56**)	114	**2.24 (1.87–2.7**)	41	0.42 (0.31–0.57)
Eye disorders	1215	0.63 (0.59–0.67)	179	0.44 (0.38–0.51)	292	0.38 (0.34–0.42)
Gastrointestinal disorders	9331	**1.16 (1.14–1.19**)	1641	0.96 (0.91–1.01)	7188	**2.50 (2.43–2.56**)
General disorders and administration site conditions	13,324	0.77 (0.76–0.78)	1998	0.53 (0.50–0.55)	5593	0.82 (0.79–0.84)
Hepatobiliary disorders	1965	**2.28 (2.18–2.39**)	633	**3.55 (3.28–3.84**)	772	**2.24 (2.09–2.41**)
Immune system disorders	2356	**2.25 (2.16–2.35**)	178	0.80 (0.69–0.92)	285	0.67 (0.60–0.75)
Infections and infestations	4750	0.94 (0.91–0.97)	2477	**2.54 (2.43–2.65**)	2367	**1.19 (1.14–1.24**)
Injury, poisoning and procedural complications	4098	0.43 (0.42–0.44)	760	0.38 (0.35–0.41)	1882	0.50 (0.48–0.52)
Investigations	5652	0.95 (0.93–0.98)	1051	0.84 (0.79–0.90)	2176	0.92 (0.88–0.96)
Metabolism and nutrition disorders	2499	**1.23 (1.18–1.28**)	545	**1.28 (1.17–1.39**)	1655	**2.07 (1.97–2.18**)
Musculoskeletal and connective tissue disorders	3458	0.67 (0.65–0.70)	527	0.49 (0.45–0.53)	631	0.30 (0.28–0.33)
Neoplasms benign, malignant and unspecified (incl cysts and polyps)	3827	**1.54 (1.50–1.60**)	999	**1.94 (1.82–2.07**)	1721	**1.75 (1.67–1.84**)
Nervous system disorders	7346	0.90 (0.88–0.92)	2278	**1.39 (1.33–1.46**)	2913	0.90 (0.86–0.93)
Pregnancy, puerperium and perinatal conditions	305	0.75 (0.67–0.83)	38	0.44 (0.32–0.61)	16	0.10 (0.06–0.16)
Product issues	158	0.10 (0.09–0.12)	12	0.04 (0.02–0.07)	26	0.04 (0.03–0.06)
Psychiatric disorders	1145	0.20 (0.19–0.21)	217	0.18 (0.16–0.21)	398	0.18 (0.16–0.19)
Renal and urinary disorders	1474	0.84 (0.80–0.88)	347	0.94 (0.85–1.05)	742	**1.06 (0.99–1.14**)
Reproductive system and breast disorders	199	0.25 (0.22–0.29)	58	0.35 (0.27–0.46)	82	0.26 (0.21–0.32)
Respiratory, thoracic and mediastinal disorders	9651	**2.25 (2.20–2.30**)	1010	**1.06 (1.00–1.13**)	2079	**1.15 (1.10–1.20**)
Skin and subcutaneous tissue disorders	6394	**1.26 (1.23–1.29**)	368	0.33 (0.30–0.36)	1678	0.81 (0.77–0.85)
Social circumstances	85	0.20 (0.16–0.25)	39	0.45 (0.33–0.61)	50	0.30 (0.23–0.40)
Surgical and medical procedures	294	0.22 (0.20–0.25)	131	0.48 (0.40–0.57)	102	0.19 (0.16–0.24)
Vascular disorders	4842	**2.43 (2.36–2.51**)	405	0.94 (0.85–1.04)	1158	**1.42 (1.34–1.51**)

Bold values, *P* < .05, ROR > 1, indicate a danger signal and have statistical significance.

AEs = adverse events, PRR = proportional reporting ratio, ROR = reporting odds ratio.

Despite these drug-specific patterns, several SOCs were consistently implicated across all 3 agents, particularly blood and lymphatic system disorders, hepatobiliary disorders, metabolism and nutrition disorders, neoplasms, and respiratory disorders. This overlap underscores shared mechanisms of toxicity, while the distinct profiles of each drug highlight their unique AE spectra. Importantly, these findings align with known adverse reactions described in drug labeling information, supporting the reliability of both the dataset and the analytical approach.

### 3.3. Signal detection at the preferred term (PT) level

**Figure 3.**
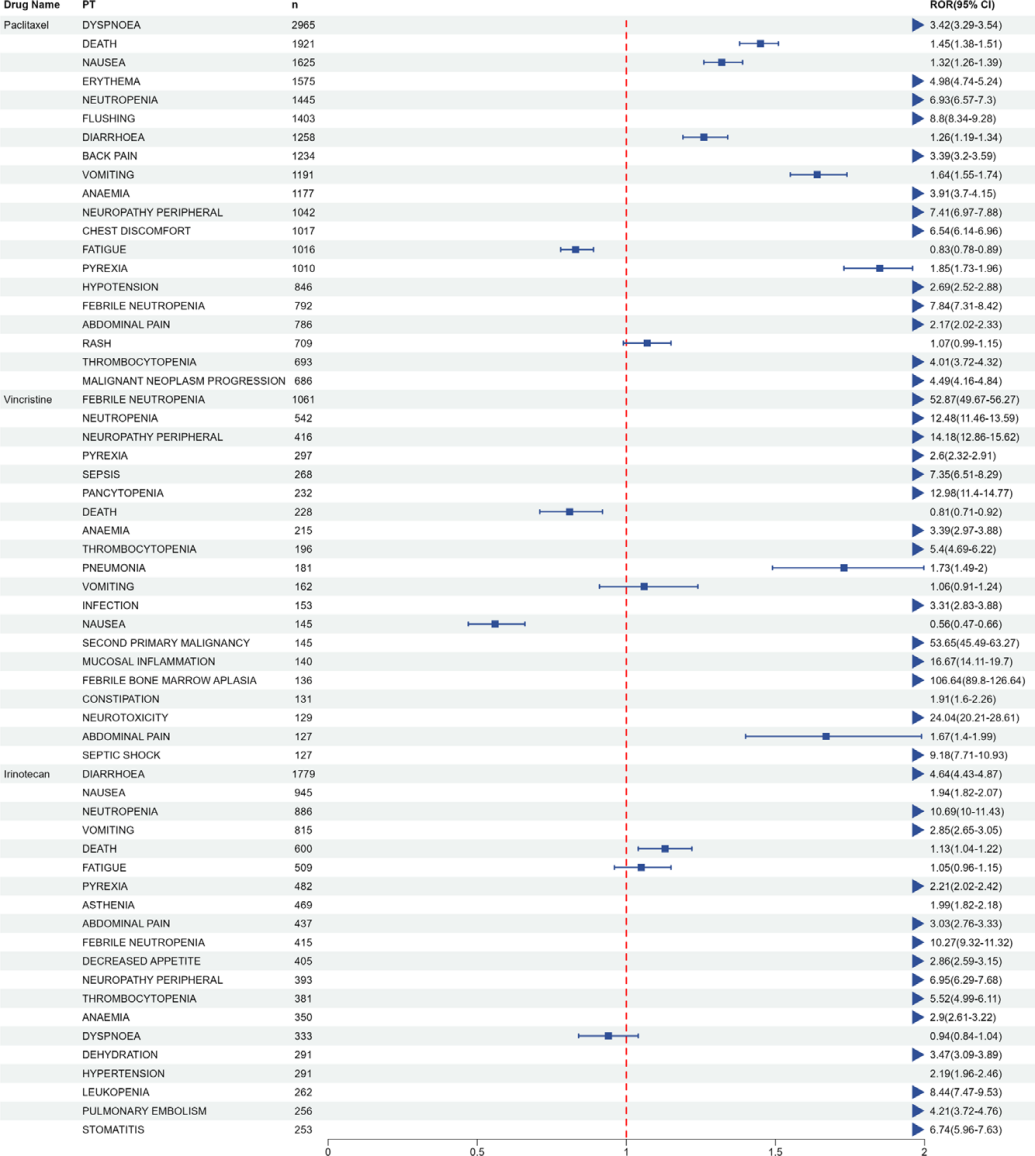
Preferred term (PT)-level signals identified using the reporting odds ratio (ROR) algorithm for paclitaxel, vincristine, and irinotecan. The top 20 signals for each drug are displayed, showing both common and drug-specific ADE profiles. ADE = adverse drug events.

We further examined ADE signals at the preferred term (PT) level. Based on the ROR algorithm, 3826 signals were identified for paclitaxel, 2329 for vincristine, and 3506 for irinotecan. As illustrated in Figure 3, we focused on the top 20 most frequently reported ADEs for each drug. According to the ROR algorithm: paclitaxel exhibited 18 significant safety signals, including dyspnea (2965), death (1921), nausea (1625), erythema (1575), neutropenia (1445), among others. Vincristine exhibited 17 significant safety signals, including febrile neutropenia (1061), neutropenia (542), peripheral neuropathy (416), pyrexia (297), sepsis (268), among others. Irinotecan exhibited 18 significant safety signals, including diarrhea (1779), nausea (945), neutropenia (886), vomiting (815), death (600), among others.

**Figure 4.**
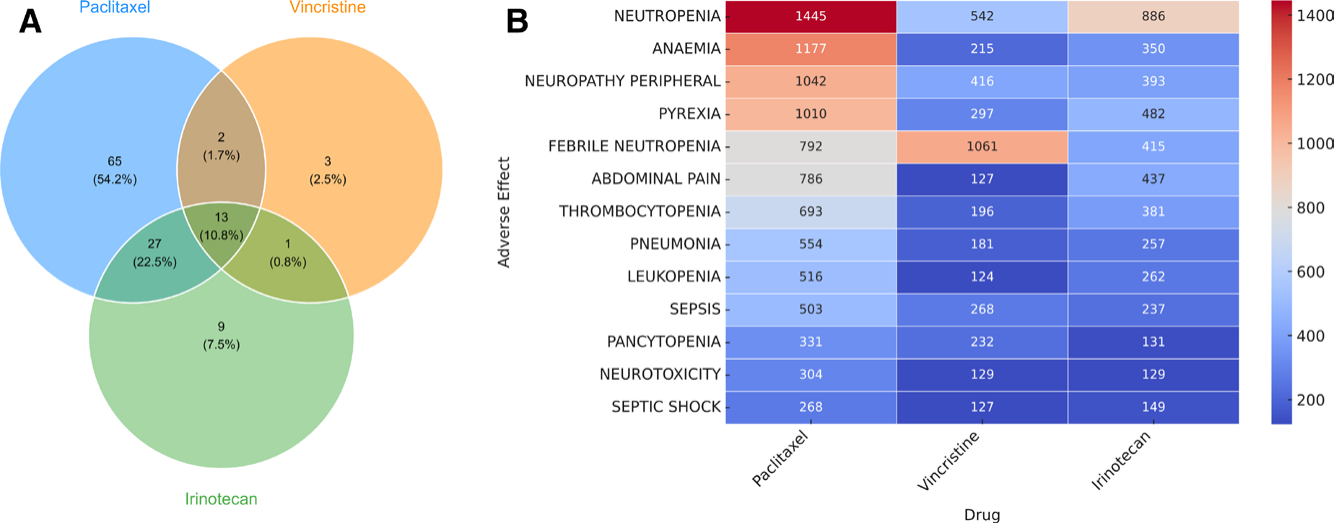
High-frequency PT signals with positive ROR values for paclitaxel, vincristine, and irinotecan. (A) Venn diagram illustrating overlapping and unique PT signals across the 3 agents. (B) Heatmap displaying the distribution and frequency of shared PT signals, highlighting neutropenia and peripheral neuropathy as common risks. PT = preferred term, ROR = reporting odds ratio.

We further analyzed PT signals that met the positive ROR threshold and had at least 100 cumulative reports. A Venn diagram (Fig. 4A) was generated to visualize the overlap of PT signals among paclitaxel, vincristine, and irinotecan. A total of 13 common PT signals (10.8%) were identified across all 3 drugs. For the intersecting PT signals, a heatmap (Fig. 4B) was constructed to examine frequency distributions. The most frequently reported ADEs were: For paclitaxel: neutropenia (1445), anemia (1177), peripheral neuropathy (1042). For vincristine: febrile neutropenia (1061), neutropenia (542), peripheral neuropathy (416). For irinotecan: neutropenia (886), pyrexia (482), abdominal pain (437). Notably, neutropenia and peripheral neuropathy appeared with high-frequency in ADE reports for all 3 drugs, suggesting these AEs may be common safety concerns for plant alkaloid-based chemotherapeutic agents.

To ensure a more rigorous evaluation of ADEs, we conducted an additional analysis of high-risk signals identified by at least one of the 4 disproportionality methods (ROR, PRR, BCPNN, MGPS). These results are summarized in Table 3. In addition to confirming known ADEs, this study identified previously unreported ADE signals: For paclitaxel, the most frequently reported ADEs included dyspnea (2965), erythema (1575), and neutropenia (1445). Additionally, 10 novel signals not previously listed in drug labeling were detected, including dyspnea, flushing, back pain, chest discomfort, and abdominal pain. For vincristine, the most frequently reported ADEs included neutropenia (1445), anemia (1177), and peripheral neuropathy (1042). Furthermore, 10 new signals were identified, including febrile neutropenia, sepsis, pancytopenia, and neoplasm progression. For irinotecan, the most frequently reported ADEs included diarrhea (1779), neutropenia (886), and vomiting (815). Additionally, 8 novel ADE signals were detected, including peripheral neuropathy, malignant neoplasm progression, dehydration, leukopenia, and pulmonary embolism.

**Table 3:** PT signals of paclitaxel, vincristine, and irinotecan, 3 plant-based chemotherapy drugs.

Drug	PT	Case numbers	ROR (95% CI)	PRR (χ^2^)	EBGM (EBGM05)	IC (IC025)
Paclitaxel	Dyspnoea*	2965	3.42 (3.29–3.54)	3.34 (4878.94)	3.33 (3.23)	1.73 (1.68)
	Erythema	1575	4.98 (4.74–5.24)	4.92 (4886.69)	4.88 (4.68)	2.29 (2.21)
	Neutropenia	1445	6.93 (6.57–7.30)	6.84 (7130.24)	6.77 (6.48)	2.76 (2.68)
	Flushing*	1403	8.80 (8.34–9.28)	8.68 (9408.4)	8.57 (8.19)	3.10 (3.02)
	Back pain*	1234	3.39 (3.20–3.59)	3.36 (2040.78)	3.35 (3.19)	1.74 (1.66)
	Anaemia	1177	3.91 (3.70–4.15)	3.88 (2505.72)	3.86 (3.68)	1.95 (1.86)
	Neuropathy peripheral	1042	7.41 (6.97–7.88)	7.34 (5642.26)	7.26 (6.90)	2.86 (2.77)
	Chest discomfort*	1017	6.54 (6.14–6.96)	6.48 (4666.7)	6.42 (6.09)	2.68 (2.59)
	Hypotension	846	2.69 (2.52–2.88)	2.68 (889.38)	2.67 (2.52)	1.42 (1.32)
	Febrile neutropenia	792	7.84 (7.31–8.42)	7.79 (4626.78)	7.70 (7.26)	2.94 (2.84)
	Abdominal pain*	786	2.17 (2.02–2.33)	2.16 (491.26)	2.16 (2.03)	1.11 (1.01)
	Thrombocytopenia*	693	4.01 (3.72–4.32)	3.98 (1541.46)	3.96 (3.72)	1.99 (1.88)
	Oxygen saturation decreased	666	7.97 (7.38–8.60)	7.92 (3974.83)	7.82 (7.34)	2.97 (2.86)
	Tachycardia*	598	4.32 (3.99–4.69)	4.30 (1507.06)	4.28 (4.00)	2.10 (1.98)
	Feeling hot*	545	5.67 (5.21–6.17)	5.64 (2062.22)	5.59 (5.21)	2.48 (2.36)
	Leukopenia	516	6.65 (6.10–7.26)	6.62 (2435.66)	6.56 (6.10)	2.71 (2.58)
	Sepsis	503	2.87 (2.63–3.13)	2.86 (605.48)	2.85 (2.65)	1.51 (1.38)
	Hyperhidrosis*	490	2.39 (2.19–2.61)	2.38 (391.85)	2.38 (2.21)	1.25 (1.12)
	Loss of consciousness*	485	2.40 (2.19–2.62)	2.39 (392.49)	2.39 (2.22)	1.26 (1.12)
	Pneumonitis	457	11.40 (10.39–12.51)	11.35 (4232.99)	11.15 (10.32)	3.48 (3.34)
Vincristine	Febrile neutropenia*	1061	52.87 (49.67–56.27)	50.09 (50181.28)	49.21 (46.7)	5.62 (5.53)
	Neutropenia*	542	12.48 (11.46–13.59)	12.16 (5541.67)	12.12 (11.28)	3.60 (3.47)
	Neuropathy peripheral	416	14.18 (12.86–15.62)	13.90 (4962.18)	13.83 (12.75)	3.79 (3.65)
	Pyrexia	297	2.60 (2.32–2.91)	2.58 (287.57)	2.57 (2.34)	1.36 (1.20)
	Sepsis*	268	7.35 (6.51–8.29)	7.26 (1445.54)	7.24 (6.55)	2.86 (2.68)
	Pancytopenia*	232	12.98 (11.4–14.77)	12.84 (2522.75)	12.78 (11.47)	3.68 (3.49)
	Anaemia	215	3.39 (2.97–3.88)	3.37 (358.63)	3.36 (3.01)	1.75 (1.55)
	Thrombocytopenia	196	5.40 (4.69–6.22)	5.36 (695.05)	5.35 (4.76)	2.42 (2.21)
	Neoplasm progression*	162	13.42 (11.50–15.68)	13.32 (1838.70)	13.26 (11.65)	3.73 (3.50)
	Infection	153	3.31 (2.83–3.88)	3.30 (244.86)	3.29 (2.88)	1.72 (1.49)
	Second primary malignancy*	145	53.65 (45.49–63.27)	53.26 (7294.78)	52.26 (45.53)	5.71 (5.47)
	Mucosal inflammation	140	16.67 (14.11–19.7)	16.56 (2035.51)	16.47 (14.32)	4.04 (3.80)
	Febrile bone marrow aplasia*	136	106.64 (89.80–126.64)	105.92 (13607.85)	102 (88.34)	6.67 (6.42)
	Neurotoxicity	129	24.04 (20.21–28.61)	23.89 (2806.09)	23.70 (20.49)	4.57 (4.31)
	Septic shock*	127	9.18 (7.71–10.93)	9.13 (916.56)	9.10 (7.86)	3.19 (2.93)
	Leukopenia	124	7.58 (6.35–9.04)	7.54 (701.75)	7.52 (6.49)	2.91 (2.65)
	Respiratory failure*	115	4.76 (3.96–5.72)	4.74 (338.90)	4.73 (4.06)	2.24 (1.97)
	Stomatitis	95	4.80 (3.93–5.88)	4.78 (284.16)	4.78 (4.04)	2.26 (1.96)
	Bone marrow failure	88	12.56 (10.18–15.49)	12.5 (927.49)	12.45 (10.45)	3.64 (3.33)
	Acute myeloid leukaemia*	88	17.52 (14.20–21.62)	17.45 (1356.25)	17.34 (14.55)	4.12 (3.81)
Irinotecan	Diarrhoea	1779	4.64 (4.43–4.87)	4.47 (4831.63)	4.46 (4.29)	2.16 (2.09)
	Neutropenia	886	10.69 (10.00–11.43)	10.47 (7547.42)	10.4 (9.83)	3.38 (3.28)
	Vomiting	815	2.85 (2.65–3.05)	2.81 (952.49)	2.8 (2.64)	1.49 (1.38)
	Pyrexia	482	2.21 (2.02–2.42)	2.19 (314.81)	2.19 (2.03)	1.13 (1.00)
	Abdominal pain	437	3.03 (2.76–3.33)	3.01 (587.98)	3.01 (2.78)	1.59 (1.45)
	Febrile neutropenia	415	10.27 (9.32–11.32)	10.17 (3410.52)	10.1 (9.32)	3.34 (3.19)
	Decreased appetite	405	2.86 (2.59–3.15)	2.84 (482.65)	2.83 (2.61)	1.50 (1.36)
	Neuropathy peripheral*	393	6.95 (6.29–7.68)	6.89 (1972.02)	6.86 (6.31)	2.78 (2.63)
	Thrombocytopenia	381	5.52 (4.99–6.11)	5.48 (1392.05)	5.46 (5.02)	2.45 (2.30)
	Malignant neoplasm progression*	368	6.03 (5.44–6.69)	5.98 (1524.03)	5.96 (5.47)	2.58 (2.43)
	Anaemia	350	2.90 (2.61–3.22)	2.88 (429.90)	2.88 (2.63)	1.52 (1.37)
	Dehydration*	291	3.47 (3.09–3.89)	3.45 (505.65)	3.44 (3.12)	1.78 (1.61)
	Leukopenia*	262	8.44 (7.47–9.53)	8.38 (1695.36)	8.34 (7.53)	3.06 (2.88)
	Pulmonary embolism*	256	4.21 (3.72–4.76)	4.19 (619.88)	4.18 (3.77)	2.06 (1.88)
	Stomatitis	253	6.74 (5.96–7.63)	6.71 (1223.97)	6.68 (6.02)	2.74 (2.56)
	Sepsis*	237	3.38 (2.98–3.85)	3.37 (394.52)	3.36 (3.02)	1.75 (1.56)
	Hypokalaemia*	203	7.12 (6.20–8.18)	7.09 (1057.85)	7.06 (6.29)	2.82 (2.62)
	Dysarthria*	197	8.33 (7.24–9.58)	8.29 (1256.13)	8.25 (7.33)	3.04 (2.84)
	Mucosal inflammation	180	11.24 (9.71–13.02)	11.19 (1658.79)	11.12 (9.83)	3.47 (3.26)
	Neutrophil count decreased	168	6.73 (5.79–7.84)	6.71 (812.90)	6.68 (5.88)	2.74 (2.52)

CI = confidence interval, EBGM = empirical Bayesian geometric mean, EBGM05 = the lower limit of the 95% CI of EBGM, IC = information component, IC025 = the lower limit of the 95% CI of the IC, PRR = proportional reporting ratio, PT = preferred term, ROR = reporting odds ratio.

* The representative explained new signals that were not mentioned.

### 3.4. Time analysis of safety signals

In this study, Table 4 presents the distribution characteristics of the time-to-onset of AEs. A total of 31,007 AE reports were associated with paclitaxel, with a median onset time of 32 days. For vincristine, 7386 AE reports were recorded, with a median onset time of 18 days. Similarly, 12,049 AE reports were linked to irinotecan, with a median onset time of 28 days.

**Table 4:** Time-to-onset of paclitaxel, vincristine, and irinotecan adverse events and Weibull distribution analysis.

Drug	TTO (d)		Weibull distribution		
	Case reports	Median (d) (IQR)	Scale parameter: α (95% CI)	Shape parameter: β (95% CI)	Type
Paclitaxel	31,007	32	61.23 (59.29~63.17)	0.64 (0.63~0.65)	Early failure
Vincristine	7386	18	46.75 (42.93~50.58)	0.54 (0.53~0.55)	Early failure
Irinotecan	12,049	28	52.99 (50.63~55.36)	0.72 (0.70~0.74)	Early failure

CI = confidence interval, IQR = interquartile range, TTO = time-to-onset.

**Figure 5.**
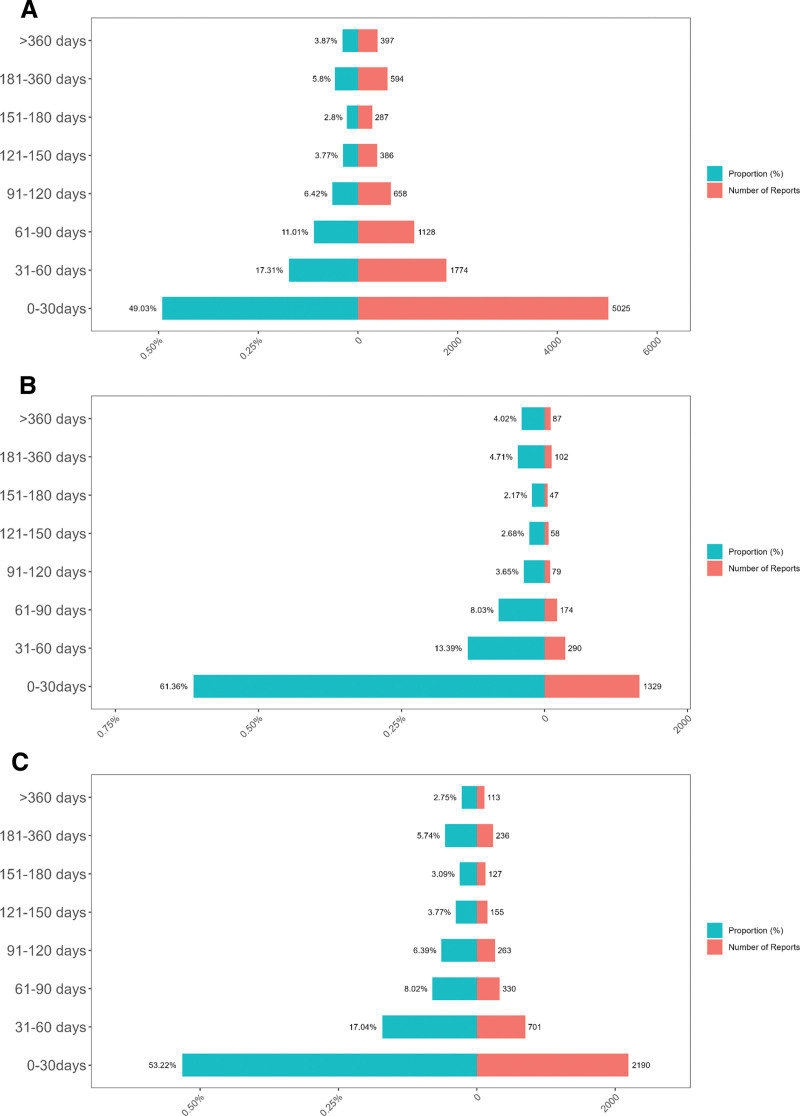
Time-to-onset distribution of ADEs associated with paclitaxel, vincristine, and irinotecan. (A) Paclitaxel, (B) vincristine, and (C) irinotecan. Most ADEs occurred within the first 30 days of treatment, showing an early failure pattern across all 3 agents. ADE = adverse drug events.

Additionally, the Weibull shape parameter (β) results indicated that for all 3 drugs, β < 1, and the corresponding 95% CI was also < 1, suggesting an early failure-type curve, where the incidence of AEs decreases over time. Analysis of the overall population distribution of time-to-onset further showed that the majority of ADEs for paclitaxel, vincristine, and irinotecan occurred within the first 0 to 30 days after drug administration (Fig. 5).

## 4. Discussion

In this study, we extracted adverse event (ADE) data for paclitaxel, vincristine, and irinotecan from the FAERS database and applied 4 disproportionality analysis methods – ROR, PRR, BCPNN, and MGPS – to comprehensively investigate potential ADE signals. The results revealed that ADE signals for these 3 drugs spanned 27 SOCs. Notably, blood and lymphatic system disorders, hepatobiliary disorders, metabolism and nutrition disorders, neoplasms (including cysts and polyps), and respiratory disorders exhibited significant signals across all 3 drugs. An important finding of this study is that over the past 5 years, the number of ADE reports for paclitaxel, vincristine, and irinotecan has shown a gradual increasing trend, suggesting that the potential risks associated with these drugs warrant further clinical attention. Furthermore, the findings of this study are highly consistent with the known adverse reactions listed in the drug labeling information, thereby confirming the effectiveness of the analytical methods used and the reliability of the data.

### 4.1. Demographic characteristics of ADEs associated with plant alkaloid-based chemotherapeutic agents

The adverse event (ADE) reports associated with paclitaxel, vincristine, and irinotecan collected in this study exhibited significant differences in gender, age, and weight distribution. For paclitaxel, the number of ADE reports from female patients was significantly higher than that from male patients (63.5% vs 26.8%). This could be attributed to the widespread use of paclitaxel in cancers with high incidence rates among women, such as breast cancer.^[14]^ In contrast, for vincristine and irinotecan, the number of reports from male patients exceeded that from female patients (vincristine: 45.9% vs 37.1%; irinotecan: 45.9% vs 33.1%). This trend may reflect the frequent use of these drugs in malignancies with higher prevalence in men, such as acute lymphoblastic leukemia and metastatic colorectal cancer.^[15,16]^ These findings suggest that sex differences in cancer epidemiology and drug utilization may directly influence the observed ADR profiles, with paclitaxel-related neurotoxicity more prominent in women and irinotecan-related gastrointestinal toxicities more frequent among men.

Regarding age distribution, the 18 to 65 year group accounted for the majority of ADEs across all 3 drugs (paclitaxel: 45.7%; vincristine: 26.7%; irinotecan: 36.7%). This trend likely reflects both the higher cancer burden and the greater capacity for chemotherapy tolerance within this age range.^[17,18]^ Notably, vincristine also demonstrated a nonnegligible proportion of reports in younger populations, consistent with its use in pediatric and adolescent leukemia. These results highlight the importance of age-tailored monitoring strategies, with younger vincristine-treated patients requiring close neurological surveillance and middle-aged or elderly irinotecan users needing careful gastrointestinal monitoring.

With respect to weight distribution, although a large proportion of cases lacked weight data (paclitaxel: 48.5%; vincristine: 72.3%; irinotecan: 65.5%), distinct patterns emerged among available data. Paclitaxel patients were primarily within the 70 to 89 kg range (45.7%), consistent with typical adult female oncology cohorts. Vincristine patients were often <50 kg (14.1%), likely reflecting its use in pediatric populations. Irinotecan patients clustered in the 50 to 69 kg range (13.8%), in line with colorectal cancer demographics. These findings emphasize that body weight is not only a demographic variable but also a surrogate for treatment indication and population characteristics, underscoring the need for more consistent weight reporting to refine pharmacovigilance assessments.^[19]^

Additionally, most ADE reports originated from developed countries such as the United States, France, Italy, Canada, and Japan (paclitaxel: United States 23.9%; vincristine: United States 23.6%, France 17.7%, Canada 15.4%; irinotecan: United States 22.0%, Japan 12.4%, France 10.6%). This distribution may be attributed to greater availability of medical resources, higher drug usage, and stronger awareness of ADE reporting in these countries.

Notably, a considerable proportion of ADE reports lacked clear indication information. For example, 23.9% of paclitaxel reports were categorized as “Product used for unknown indication,” suggesting off-label drug use or incomplete reporting. This underscores the need for more precise and standardized reporting in future pharmacovigilance monitoring.

### 4.2. SOC characteristics of ADEs

Our study identified distinct SOC patterns associated with paclitaxel, vincristine, and irinotecan, revealing their primary safety concerns.

For paclitaxel, the ADE signals were predominantly concentrated in gastrointestinal disorders, skin and subcutaneous tissue disorders, immune system disorders, blood and lymphatic system disorders, respiratory disorders, vascular disorders, cardiac disorders, metabolism and nutrition disorders, neoplasms (including cysts and polyps), hepatobiliary disorders, and endocrine disorders. In blood and lymphatic system disorders, neutropenia and thrombocytopenia were the most frequently reported ADEs, likely due to paclitaxel-induced myelosuppression.^[20]^ In immune system disorders, hypersensitivity reactions and immune-mediated toxicities were notable. In nervous system disorders, peripheral neuropathy was the most frequently reported ADE, which may be associated with paclitaxel-induced neurotoxicity.^[21]^ In gastrointestinal disorders, nausea, vomiting, and diarrhea were commonly reported, indicating a strong irritative effect on gastrointestinal mucosa. In skin and subcutaneous tissue disorders, rash and alopecia were among the most frequently reported ADEs, possibly due to paclitaxel-induced toxicity to epidermal cells.^[22]^

For vincristine, the ADE signals were mainly observed in congenital, familial, and genetic disorders, blood and lymphatic system disorders, nervous system disorders, metabolism and nutrition disorders, infections and infestations, hepatobiliary disorders, neoplasms (including cysts and polyps), ear and labyrinth disorders, and endocrine disorders. In blood and lymphatic system disorders, thrombocytopenia and leukopenia were the most commonly reported ADEs, likely resulting from vincristine-induced bone marrow suppression.^[23]^ In nervous system disorders, peripheral neuropathy was the most frequently observed ADE, characterized by paresthesia and motor dysfunction, which is associated with vincristine-induced neurotoxicity.^[24]^ In gastrointestinal disorders, nausea, vomiting, and constipation were frequently reported, which may be attributed to vincristine’s inhibitory effects on gastrointestinal smooth muscle.^[25]^ Regarding infections and infestations, vincristine was found to increase infection risk, potentially due to its immunosuppressive effects.^[26]^

For irinotecan, the ADE signals were predominantly associated with gastrointestinal disorders, infections and infestations, respiratory disorders, blood and lymphatic system disorders, metabolism and nutrition disorders, hepatobiliary disorders, neoplasms (including cysts and polyps), and vascular disorders. In blood and lymphatic system disorders, neutropenia and thrombocytopenia were the most frequently reported ADEs, which may be attributed to irinotecan-induced bone marrow suppression.^[27]^ In gastrointestinal disorders, diarrhea was the most commonly reported ADE, likely due to irinotecan’s toxic effects on intestinal mucosa.^[28]^ In hepatobiliary disorders, liver function abnormalities were frequently reported, possibly related to irinotecan-induced hepatocellular toxicity.^[29]^ Additionally, irinotecan was found to increase the risk of infections, potentially due to its immunosuppressive effects.^[30]^ These findings provide valuable insights into the specific ADE profiles of paclitaxel, vincristine, and irinotecan, which can aid in risk assessment and clinical decision-making.

### 4.3. Novel ADEs associated with plant alkaloid-based chemotherapeutic agents

In this study, we utilized 4 disproportionality analysis methods – ROR, PRR, BCPNN, and MGPS – to further explore adverse event (ADE) data associated with paclitaxel, vincristine, and irinotecan, with a particular focus on previously unreported ADE signals that were not included in the drug labeling.

For paclitaxel, we identified 10 novel ADE signals, including dyspnea (respiratory distress), flushing, back pain, chest discomfort, and abdominal pain. These newly detected signals suggest that paclitaxel may exert additional toxic effects on the respiratory and cardiovascular systems, particularly in relation to dyspnea and flushing, which could be linked to direct drug toxicity or immune responses.^[31,32]^

For vincristine, we identified 10 novel ADE signals, including febrile neutropenia, sepsis, pancytopenia, and neoplasm progression. These signals indicate that vincristine may pose additional risks related to immunosuppression and tumor progression, highlighting the necessity of close monitoring of hematologic parameters and infection risks during clinical use.

For irinotecan, we identified 8 novel ADE signals, including peripheral neuropathy, malignant neoplasm progression, dehydration, leukopenia, and pulmonary embolism. These findings suggest that irinotecan may have additional toxic effects on the nervous, hematologic, and respiratory systems, particularly concerning peripheral neuropathy and pulmonary embolism, which warrant increased clinical vigilance.

Overall, this study identified multiple novel ADE signals for paclitaxel, vincristine, and irinotecan, which have not been previously documented in drug labeling but could significantly impact patient outcomes. These findings provide critical safety insights for clinical practice, emphasizing the need for enhanced monitoring and management of these ADEs to ensure patient safety.

### 4.4. Time-to-onset analysis of ADEs associated with plant alkaloid-based chemotherapeutic agents

This study further analyzed the time-to-onset distribution of AEs (ADEs) associated with paclitaxel, vincristine, and irinotecan to assess their potential safety risks. The results showed that paclitaxel had a median time-to-onset of 32 days (31,007 reports), vincristine had a median onset time of 18 days (7386 reports), and irinotecan had a median onset time of 28 days (12,049 reports). These findings suggest that while ADEs associated with these 3 drugs occur at different time points, vincristine-induced ADEs tend to occur earlier, whereas paclitaxel-related ADEs appear relatively later.

Additionally, Weibull survival probability analysis revealed that the shape parameter (β) for all 3 drugs was <1, with a 95% CI also below 1, indicating a decreasing incidence of ADEs over time and an early failure-type curve pattern. Population-level time distribution analysis further showed that the majority of ADEs for all 3 drugs occurred within the first 0 to 30 days after chemotherapy initiation, suggesting an elevated risk of ADEs during the early phase of treatment.^[33]^

These findings highlight that ADEs associated with plant alkaloid-based chemotherapeutic agents predominantly occur during the early stages of treatment, with a declining trend over time. Consequently, intensified patient monitoring and management should be prioritized within the first 30 days of chemotherapy, ensuring timely identification and intervention for potential ADEs. Moreover, the early failure-type curve pattern underscores the importance of enhanced pharmacovigilance during the initial treatment phase, providing valuable time-based risk assessment for clinical decision-making.

### 4.5. Limitations

This study leveraged large-scale real-world data from the FAERS database and employed multiple disproportionality analysis methods to investigate the safety signals of plant alkaloid-based chemotherapeutic agents. However, several limitations remain.

First, the FAERS database relies on a voluntary reporting mechanism, which may lead to underreporting or overreporting of ADEs. Additionally, the data quality varies across different reporting sources, potentially affecting the accuracy of signal detection. Second, detailed clinical information of individual patients, such as medical history, concomitant medications, and treatment dosage, is often missing in the FAERS database. This limitation hinders the control of potential confounding factors, making it difficult to establish a definitive causal relationship between ADEs and specific drugs.

Moreover, the disproportionality analysis methods (ROR, PRR, BCPNN, and MGPS) used in this study can only detect statistical associations rather than causal relationships. The occurrence of ADE signals may be influenced by individual patient variability, underlying diseases, or concomitant drug use. Therefore, further clinical studies and prospective cohort research are needed to validate the ADE signals identified in this study.

## 5. Conclusion

This study systematically analyzed ADE signals of paclitaxel, vincristine, and irinotecan using multiple disproportionality analysis methods based on the FAERS database. The results revealed potential safety risks across several SOCs, particularly hematologic, gastrointestinal, neurological, and immune system disorders. Beyond confirming well-established toxicities, our analysis also identified previously unreported ADE signals, thereby expanding the safety profile of these plant alkaloid–derived chemotherapeutic agents and providing important evidence for pharmacovigilance.

Importantly, our findings highlight demographic-specific differences, with sex, age, and body weight distributions shaping distinct ADE patterns. These insights provide a basis for personalized risk stratification, such as closer monitoring of neurotoxicity in female patients receiving paclitaxel or gastrointestinal complications in male patients treated with irinotecan. Such targeted surveillance strategies could help clinicians anticipate adverse outcomes and improve real-world treatment safety.

While the use of large-scale real-world data enhances robustness, limitations remain, including underreporting, incomplete patient information, and the inability to confirm causality. Future research should integrate pharmacovigilance data with prospective clinical studies, mechanistic experiments, and genomic profiling to validate signals and unravel underlying biological pathways. Ultimately, strengthening adverse event reporting systems and advancing precision medicine approaches will support safer and more effective use of plant alkaloid-based chemotherapy.

## Acknowledgments

This study was performed using open-source data provided by the FAERS database, and we thank all those who provided information for this database.

## Author contributions

**Funding acquisition:** Jihong Hu.

**Methodology:** Bangguo Song, Yang Zhang, Li Wang, Jihong Hu.

**Software:** Bangguo Song, Yang Zhang, Li Wang.

**Supervision:** Jihong Hu.

**Validation:** Yang Zhang.

**Visualization:** Yang Zhang.

**Writing – original draft:** Yang Zhang, Jihong Hu.

**Writing – review & editing:** Bangguo Song, Yang Zhang, Li Wang, Jihong Hu.

## Supplementary Material


