# Supplementary material for: Comprehensive pharmacovigilance of phytoalkaloid chemotherapeutics: Signal detection and time-to-onset analysis based on FAERS
Source: Medicine (Baltimore). 2025 Nov 21;104(47):e46044. doi: 10.1097/MD.0000000000046044 (PMC12643699; doi:10.1097/MD.0000000000046044)
Supplement: Supplementary file 1 [file medi-104-e46044-s001.pdf]

Supplementary Material

Two-by-two contingency table for disproportionality analyses.

Drugs	Target AEs	Other AEs	Total
Target drugs	a	b	a+b
Other drugs	c	d	c+d
Total	a+c	b+d	a+b+c+d

Abbreviation: AEs, adverse events; a, number of reports containing both the target drug and target adverse drug reaction; b, number of reports containing other adverse drug reaction of the target drug; c, number of reports containing the target adverse drug reaction of other drugs; d, number of reports containing other drugs and other adverse drug reactions.

Four major algorithms used for signal detection.

Algorithms	Equation	Criteria
ROR	ROR=ad/b/c<br>95%CI=$e^{\ln(ROR)\pm 1.96(1/a+1/b+1/c+1/d)^{\wedge}0.5}$	lower limit of 95% CI>1, N ⩾3
PRR	PRR=a(c+d)/c/(a+b)<br>χ^2=[(ad-bc)^2](a+b+c+d)/[(a+b)(c+d)(a+c)(b+d)]	PRR⩾2, χ^2⩾4, N⩾3
BCPNN	IC=$\log_2$a(a+b+c+d)(a+c)(a+b)<br>95%CI= E(IC) ± 2V(IC)^0.5	IC025>0
EBGM	EBGM=a(a+b+c+d)/(a+c)/(a+b)<br>95%CI=$e^{\ln(EBGM)\pm 1.96(1/a+1/b+1/c+1/d)^{\wedge}0.5}$	EBGM05>2

Abbreviation: a, number of reports containing both the target drug and target adverse drug reaction; b, number of reports containing other adverse drug reaction of the target drug; c, number of reports containing the target adverse drug reaction of other drugs; d, number of reports containing other drugs and other adverse drug reactions. 95%CI, 95% confidence interval; N, the number of reports; χ2, chi-squared; IC, information component; IC025, the lower limit of 95% CI of the IC; E(IC), the IC expectations; V(IC), the variance of IC; EBGM, empirical Bayesian geometric mean; EBGM05, the lower limit of 95% CI of EBGM.
